# The causal relationship between smoking, alcohol consumption, and renal clear cell carcinoma: a Mendelian randomization study

**DOI:** 10.3389/fgene.2024.1391542

**Published:** 2024-06-18

**Authors:** Hongbin Cui, Junji Du, Hongbo Xue, Yingjian Zhao, Chengwen Li

**Affiliations:** ^1^ Tianjin Medical University, Tianjin, China; ^2^ Tianjin Fourth Central Hospital, Tianjin, China

**Keywords:** smoke, alchohol, Mendelian randomization, renal cell caecinoma, causal relationship

## Abstract

**Introduction:** Observational studies have found a correlation between the consumption of tobacco and alcohol and the likelihood of developing renal cell carcinoma. However, whether these associations indicate causal relationships is unclear.

**Methods:** To establish if these connections indicate causal relationships, we performed a Mendelian Randomization (MR) analysis using a two-sample approach. For the number of daily cigarettes, lifetime smoking index, smoking initiation, and weekly drinking, we employed 44, 108, 174, and 76 single nucleotide polymorphisms (SNPs) as instrumental variables. Outcome data were obtained from the FinnGen Alliance, which included a combined total of 429,290 individuals. The MR analysis was conducted using the inverse-variance weighted (IVW) method to estimate causal effects. To address potential violations of MR assumptions due to directional pleiotropy, we performed MR-Egger regression and MR-PRESSO (Mendelian Randomization Pleiotropy RESidual Sum and Outlier) analysis.

**Results:** Genetically influenced smoking initiation was directly associated with the risk of developing renal cell carcinoma (OR = 1.55, 95% CI: 1.04–2.33; *p* = 0.03). No causal relationship was found between daily cigarette consumption and lifetime smoking index with the risk of renal cell cancer. Genetic predisposition for weekly alcohol consumption showed a reduced risk of renal cell cancer (OR = 0.45, 95% CI: 0.26–0.81; *p* = 0.007).

**Discussion:** Our study suggests a potential causal relationship between alcohol consumption and reduced risk of renal cell cancer, while no such association was observed with smoking. Further research is needed to confirm these findings.

## 1 Introduction

Over the last couple of years, there has been a notable rise in the prevalence of renal cell carcinoma (RCC), with an annual increase of around 1.1%. (Palumbo et al., 2021). Compared to 1990, there was a 154.78% increase in RCC cases by 2019. (Zi et al., 2021). During the three-decade study duration, there was a yearly rise in the age-adjusted mortality rate and age-adjusted rate of RCC, along with the disability-adjusted life rate (estimated annual percentage changes = 0.35 and 0.12). (Zi et al., 2021). Previous studies have identified several risk factors for RCC, but the accurate pathogenesis remains unclear. (Capitanio et al., 2019; Yuan et al., 2021). Since RCC is a deadly condition, it is imperative to identify intervention strategies that can decrease the likelihood of developing this illness.

Smoking and drinking are health risk factors. Many studies have shown that smoking is a risk factor for kidney cancer, while alcohol consumption has a protective effect against the occurrence of kidney cancer. (Capitanio et al., 2019; Bukavina et al., 2022; Kim et al., 2023). However, the nature of the relationship between smoking, drinking, and the risk of RCC remains uncertain. Moreover, the outcomes of current observational epidemiological studies could be impacted by combined and reciprocal causality, which holds significant importance for implementing clinical intervention strategies and developing recommendations for public policy. While a randomized controlled trial (RCT) is the optimal approach to deduce causation, (Bhide et al., 2018), it is impractical and morally wrong to carry out an RCT to evaluate the influence of smoking and alcohol intake on RCC. In observational epidemiological research, Mendelian randomization (MR) employs single nucleotide polymorphisms (SNPs) as instrumental variables to evaluate the effects of the outcomes of interest and mitigate bias. Theoretically, MR and RCT share similarities and random distribution occurs in the assignment. The impact of reverse causality on MR is minimized due to the fact that genetic variation remains constant from conception. Furthermore, MR is more resistant to environmental confounding in contrast to conventional observational research. The reason for this is that in MR, it is assumed that genetic instrumental variables (IVs) only impact the outcomes through exposure and are not related to any confounding factors. (Hemani et al., 2018a; Saunders et al., 2022).

To investigate the impact of genetics on the susceptibility to kidney cell carcinoma and its potential causal association with alcohol consumption, we employed a two-sample MR analysis, focusing on a single variable.

## 2 Materials and methods

### 2.1 MR design

**FIGURE 1:**
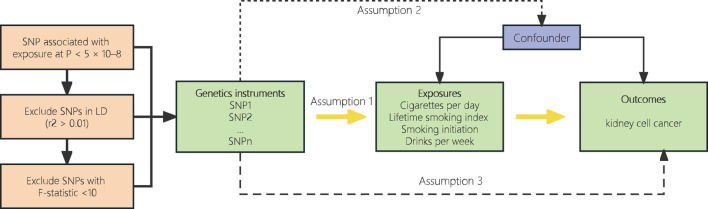
Overview and assumptions of the Mendelian randomization study design.

To be considered valid instrumental variables, genetic variants must meet three essential criteria: 1) they must exhibit a strong correlation with the exposure; 2) they should not be linked to any potential confounding factors associated with the exposure outcomes; and 3) besides the exposure, they should not have any impact on the outcomes of other variables (Figure 1). (Hemani et al., 2018a)

### 2.2 Selection of instrument variables

To fulfill the initial requirement of Mendelian randomization (MR), uncorrelated individual nucleotide variations (SNPs) linked to the exposure at the genome-wide significance threshold (*p* < 5 × 10^−8^) and free from genetic linkage (*r* ^2^ < 0.01 and cluster window >10,000 kb) were utilized as instrumental factors (IVs). (Didelez and Sheehan, 2007). To validate the initial hypothesis, we computed the ratio of phenotypic variability elucidated by the complete collection of SNPs and the F statistic. (Burgess et al., 2016a). The strength of the instrument depends on the precision and extent to which the IV is associated with the risk factor. If the associated F statistic exceeds 10, it is deemed satisfactory.

### 2.3 Sources of exposure data

We acquired the summary-level data for the entire genetic makeup (Supplementary Table S1). (Saunders et al., 2022). To ensure the uniformity of the sample, we specifically excluded individuals of non-European descent for the two sample MR analysis. (Hemani et al., 2018a). The SNPs of daily cigarettes was obtained from the GWAS and Sequencing Consortium of Alcohol and Nicotine Use (GSCAN), encompassing 784,353 individuals. It is defined as the average daily smoking among both current and former smokers. (Saunders et al., 2022). Data from the GWAS abstract were used to obtain the lifetime smoking index. (Wootton et al., 2020). This encompasses information about smoking intensity, duration, and smoking cessation, which are amalgamated into a comprehensive lifelong smoking index. The GWAS researcher has created a framework that includes time, duration of smoking, daily number of cigarettes, and fixed values for decay rate and delay time to comprehend the non-linear hazards linked to smoking. (Wootton et al., 2020). After excluding individuals who lack phenotype data and have not been eliminated by genotype, GWAS still has 462,690 participants. In addition, the SNP used for smoking initiation was derived from GSCAN, involving a total of 3,383,199 European blood participants, representing the likelihood of regular smoking. (Saunders et al., 2022). We obtained the GWAS data for weekly drinking from the GSCAN, which included data from 2,965,643 individuals. (Saunders et al., 2022). The weekly intake of alcoholic drinks by the participants in the study is measured, including a variety of alcoholic beverages.

### 2.4 Sources of outcome data

We obtained the summary data of the genetic correlation with kidney cell carcinoma, its subtypes, and urothelial carcinoma from the FinnGen League (Supplementary Table S1). The Finnish consortium has recently released the R10 dataset, comprising a grand total of 429,290 individuals of Finnish origin, both male and female. Individuals with excessive hybridization, lacking high-quality genotypes, ambiguous gender, and non-Finnish blood were excluded (https://finngen.gitbook.io/documentation/). The size of all genetic correlation effects is calculated by logical regression and adjusts ages, gender, and genetic main components.

### 2.5 Statistical analyses

In order to examine the third MR hypothesis, we assessed the heterogeneity of independent SNP effects by employing the Cochran Q statistic and the MR Egger intercept test to detect directional pleiotropy. (Hemani et al., 2018b; Bowden et al., 2018). After the MR-Egger regression adjusts for pleiotropy, it can provide estimated values, but the statistical power is reduced. The MR-Egger intercept’s *p*-value determines the presence of directional pleiotropy. The primary statistical analysis method employed was the inverse-variance weighted (IVW) approach. In cases where horizontal pleiotropy is either equalized or absent, the IVW technique can provide an impartial estimation. (Hemani et al., 2018b). The genome-wide association studies (GWAS) utilized individuals of exclusively European descent, and the genetic principal component was employed to account for population structure in these GWAS. To address pleiotropy, we utilized various methods including MR-PRESSO, penalized weighted median, IVW radial regression, and weighted median as robust approaches. (Hemani et al., 2018b). The objective of the MR-PRESSO approach is to identify possible anomalies and produce estimates once they are eliminated. Distortion tests are used to detect discrepancies between estimates before and following the elimination of outliers. Assuming that the estimated weight of the MR effect is not more than 50% of the pleiotropic SNP effect, the weighted median method penalizes it to provide a consistent effect estimation. The weight depends on the strength of its correlation with the exposure. IVW radial regression employs an enhanced second-order weight to test and remove peripheral SNPs. At least 50% of the specified analysis in the weighted median comes from effective instrumental variables, and the weighted mode, which necessitates the largest subset to determine the same causal effect, constitutes effective tools. (Hartwig et al., 2017). Statistical analyses were conducted utilizing the R software, version 4.3.2, with the TwoSampleMR (0.5.8) and MR-PRESSO (1.0) packages.

## 3 Results

### 3.1 Selection of genetic instruments and Calculation of F-statistics

For daily cigarettes, lifetime smoking index, smoking initiation, and drinks per week, instrumental variables (IVs) consist of 44, 108, 174, and 76 SNPs in total. The minimum F-statistics are 25.50, 31.36, 20.29, and 22.61.

### 3.2 The identification of diversity, one-way pleiotropy, and exceptional cases

Supplementary Table S1 presents the units for RCC. Supplementary Table S2 provides a description of the phenotype in the genome-wide association (GWAS) study of exposure and its outcomes. To summarize, the examination shows no variation when considering smoking and drinking as factors, and the MR-Egger intercept analysis does not detect any biased effects in any of the assessments (Supplementary Table S3). MR-PRESSO analysis did not detect any abnormal values (Supplementary Table S4).

### 3.3 Univariate MR analysis

**FIGURE 2:**
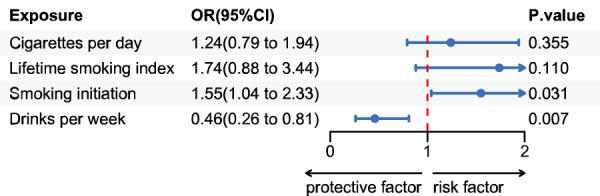
Estimates for the association of genetic liability for cigarettes per day, lifetime smoking index, smoking initiation and drinks per week with risk of renal cell cancer. Odds ratios per SD increment in the exposure from single-variable inverse variance weighted analysis.

The FinnGen consortium study found that individuals with a genetic inclination towards starting smoking had a higher likelihood of developing renal cell cancer, as suggested by multiple estimates accompanied by wider confidence intervals (CIs) (Figure 2).

With a one-standard deviation increase in the number of smoking initiations, the odds ratio (OR) for kidney cell cancer exhibited a value of 1.55 (IVW 95% CI 1.04–2.33; *p* = 0.03) and 2.03 (WM 95% CI 1.11–3.72; *p* = 0.02). While not statistically significant in the MR-Egger regression analysis, this causal relationship remained relatively consistent in the supplementary analysis (Supplementary Figure S1). There is no potential causal link between the anticipated number of cigarettes smoked daily based on genetics and the lifetime smoking index with RCC. The odds ratio (OR) is 1.24 (IVW 95% CI: 0.79–1.93; *p* = 0.35) and 1.74 (IVW 95% CI: 0.88–3.43; *p* = 0.11), respectively. These non-significant associations remain consistent in the supplementary analysis (Supplementary Figure S1). Genetic predisposition to drinks per week demonstrated a protective effect against kidney cell cancer. The odds ratio (OR) for kidney cell cancer decreased to 0.45 (IVW 95% CI 0.26–0.81; *p* = 0.007) with a weekly increase of one standard deviation in the number of alcoholic beverages consumed. This association remained almost consistent in supplementary analyses (Supplementary Figure S1).

## 4 Discussions

Using two-sample Mendelian randomization (MR), we assessed the potential relationship between the risk of smoking and drinking and RCC. A positive relationship was observed between the initiation of smoking and the risk of kidney cell carcinoma, although no significant association statistics were found for daily cigarette consumption and the lifelong smoking index. This suggests a potential risk discrepancy among different exposure levels. Therefore, we cannot conclusively determine the association between smoking and kidney cell carcinoma. Additionally, we identified drinking as a protective factor against RCC. Across various exposure and analysis models, the MR estimation results were consistent in both size and direction.

Previous observational studies have shown that smoking was a major contributing factor to the development of RCC. (Bukavina et al., 2022; Li and Hecht, 2022; Campi et al., 2023). The majority of research indicates that individuals who smoke are twice as likely to develop renal cell cancer. The incidence of RCC appears to increase exponentially in people who smoke for more than 22 pack-years. (Campi et al., 2023). After conducting a comprehensive analysis of 56 research papers, it has been determined that individuals who currently engage in smoking are subjected to a 39% elevated likelihood of developing kidney cancer (KC). The authors also reported a 20% higher risk for former smokers and a 26% higher risk for ever-smokers compared to never-smokers. The correlation between smoking and the risk of KC is directly proportional to the amount smoked, with a significant rise in risk for individuals who smoke up to 30 cigarettes daily. For individuals who smoked 5, 10, 20, and 30 cigarettes per day, the relative risk (RR) was 1.18 (95% CI 1.22–1.52), 1.61 (95% CI 1.40–1.86), and 1.72 (95% CI 1.52–1.92), respectively. (Liu et al., 2019). The likelihood of developing kidney cancer (KC) decreases steadily over time after quitting smoking cigarettes. At 10, 20, and 30 years after quitting, the risk ratio (RR) for individuals who used to smoke compared to those who currently smoke is 0.94 (95% CI 0.87–1.01), 0.88 (95% CI 0.76–1.02), and 0.82 (95% CI 0.66–1.02) correspondingly. (Liu et al., 2019). The exact mechanism of increasing RCC (RCC) risk has not yet been fully understood. Tobacco contains a large number of chemicals, with over 50 of them classified as human carcinogens. (Li and Hecht, 2022). The mutagenic activity of tobacco carcinogens can be attributed to smoke, which serves as a risk factor for RCC. These carcinogens are converted into reactive metabolic products, which can combine with DNA and cause structural changes. (Buendia Jimenez et al., 2015). ANKS1B is a gene that encodes the primary form of a protein involved in transmitting signals through tyrosine kinase pathways, characterized by the presence of an Ankyrin repeat and sterile alpha motif domain. Compared with the patient-matched normal kidney tissue, ANKS1B is expressed at a decreased level in the kidney tumor tissue of RCC patients. (Eckel-Passow et al., 2014). A single nucleotide polymorphism in ANKS1B was found in the tissue from patients with lung cancer, and a correlation with carcinogenic metabolism was established, (Lin et al., 2012), which indicates that ANKS1B may have a similar effect in RCC and plays potential tumor inhibitory Gene. Polymorphism in other genes, such as N-acetyl metastases 2 (NAT2) and glutathione S-metastases (GSTM1) is also related to the increased cancer risk of RCC in the smokers. Compared to individuals with a fast NAT2 acetyl gene or GSTM1-positive genotype, those with a slow NAT2 acetyl genome or GSTM1-NULL genome have a higher risk of RCC. (Cumberbatch et al., 2016). NAT2 and GSTM1 play a role in the breakdown and elimination of cancer-causing substances present in tobacco, thus forming the basis of this connection. Individuals with a slow NAT2 acetyl genome and GSTM1-NULL genotype exhibit impaired metabolic ability, leading to an increased risk of these cancers. (Cumberbatch et al., 2016).

Our discovery regarding the association between drinking and RCC is in line with previous observational research. Several potential studies have suggested that the consumption of alcohol in small to moderate amounts has a dose-dependent protective impact on the progression of RCC. (Lew et al., 2011; Minami et al., 2021; van de Pol et al., 2021). Typically, the intake of a minimum of 15 g of alcohol daily is linked to a lower chance of RCC, with an estimated reduction of 28%. (Cumberbatch et al., 2016). Bagnardi et al. conducted a comprehensive meta-analysis. (Bagnardi et al., 2015). They observed a statistical inverse correlation between RCC and alcohol consumption. According to the researchers, in a total of 24 studies, there is a decreased risk indicated by a relative risk (RR) of 0.92 (95% CI 0.86–0.99), specifically linked to moderate alcohol consumption. Several mechanisms have been proposed by which alcohol consumption might reduce the risk of RCC. First, light to moderate alcohol consumption has been reported to enhance insulin sensitivity, (Davies et al., 2002; Bonnet et al., 2012; Schrieks et al., 2015), which may play a role in diabetes management. Given that a meta-analysis has shown a significant positive association between diabetes and RCC incidence, improved insulin sensitivity through alcohol consumption could serve as an indirect protective factor against RCC. (Larsson and Wolk, 2011). Second, alcoholic beverages contain antioxidant phenolic compounds that can reduce oxidative stress. These compounds help to remove oxidized carcinogenic agents, reduce lipid peroxidation and cell proliferation, and promote apoptosis. (Gago-Dominguez et al., 2002; Lee et al., 2007). Third, alcohol’s diuretic effect might contribute to controlling hypertension, a known risk factor for RCC. (Lee et al., 2007). However, it is important to note that an increase in total fluid intake has not been conclusively established to influence RCC risk. (Lee et al., 2007; Hu et al., 2009).

There are several advantages in our research. The primary advantage is the implementation of Mendelian Randomization (MR) design, which helps in minimizing residual confounding factors and reverse causal relationships. Additionally, our study is conducted using a substantial number of RCC cases from two independent research groups. The entire analysis is restricted to individuals of European ancestry, aiming to minimize the potential for stratification bias within the population. To minimize the chance of instrumental weakness, we implemented rigorous selection criteria (*p* < 5 × 10^−8^) when choosing instruments. Furthermore, all related F-statistics surpassed 10, suggesting that the genetic instruments employed exhibit robust strength and are not affected by biases caused by weaker instruments. (Burgess et al., 2016b). In our research, sensitivity analysis conducted through the MR-Egger model reveals no evidence of horizontal pleiotropy in single-variable analysis, suggesting the validity of our results. To evaluate and reduce heterogeneity and multifactorial effects, we conducted a sensitivity analysis.

Our research has some limitations. First, the standard Mendelian Randomization (MR) assumes a linear relationship between risk factors and outcomes. In cases where the real correlation is not linear, the measured estimation value can be deceptive, despite still indicating the presence and direction of the average causal impact within the group. (Burgess and Thompson, 2015). Secondly, in our research, the instrumental variables IV) for smoking and drinking may be associated with other risk factors for kidney cell carcinoma. Therefore, the relationship between genetic mutations and kidney cell carcinoma may be easily influenced by these factors. Third, exposure assessment relies on self-reported information, making it prone to underestimation. The inherent measurement error in exposure assessment does not impact the instrumental variable analysis. Fourth, the impact of different types of cigarettes or alcohol on smoking and drinking can vary. Furthermore, it is important to exercise caution prior to summarizing the results. Moreover, it is crucial to prioritize the discovery and advancement of novel biomarkers, alongside additional risk factors, that have the ability to identify and forecast clinical results in individuals diagnosed with RCC.

## 5 Conclusions

Our MR study demonstrated that alcohol consumption can lower the risk of RCC, but there is no compelling evidence to suggest a causal relationship between smoking and the risk of RCC. Further longitudinal and experimental studies are still required to validate our findings.

## Data Availability

The data presented in the study are deposited in the Data Repository for the University of Minnesota (DRUM), DOI: https://doi.org/10.13020/przg-dp88; University of Bristol Research Data Repository, DOI: 10.5523/bris.10i96zb8gm0j81yz0q6ztei23d and Finngen R10 Study, https://r10.risteys.finngen.fi/endpoints/C3_KIDNEY_NOTRENALPELVIS_EXALLC.
