# Supplementary material for: The causal relationship between smoking, alcohol consumption, and renal clear cell carcinoma: a Mendelian randomization study
Source: Front Genet. 2024 Jun 18;15:1391542. doi: 10.3389/fgene.2024.1391542 (PMC11217166; doi:10.3389/fgene.2024.1391542)
Supplement: Supplementary file 2 [file Table1.DOCX]

Supplementary Table 1 Information on genetic instruments and outcome data sources

| Exposures | Consortium or cohort study | Participants | Adjustments | Data sources |
| --- | --- | --- | --- | --- |
| Cigarettes per day | GWAS and Sequencing Consortium of Alcohol and Nicotine Use (GSCAN) | 784,353 European-descent individuals | Age, sex, and the first 10 genetic principal  components | https://genome.psych.umn.edu/index.php/GSCAN  PubMed ID: 36477530 |
|  |  |  |  |  |
| Smoking initiation |  | 3,383,199 European-descent individuals |  |  |
| Drink per week |  | 2,965,643 European-descent individuals |  |  |
| Life smoking index | UK biobank | 462,690 European-descent individuals | Accounts for population stratification and  relatedness using linear mixed modelling.  Genotyping chip and sex were included as  covariates | https://data.bris.ac.uk/data/datasda/10i96zb8gm0j81yz0q6ztei23  PubMed ID: 31689377 |
| Kidney cell cancer | FinnGen | 429,209 European-descent individuals | After the removal of individuals with excess heterozygosity (±4 SD), high genotype missingness (>5%), ambiguous gender, and non-Finnish ancestry. All genetic association effect sizes were computed by multivariable logistic regression and adjusted for sex, age, and genetic principal components. | https://www.finngen.fi/fi |

Supplementary Table 3. Heterogeneity of Wald ratios and MR-Egger test for directional pleiotropy

| Exposure | Heterogeneity | |  | MR-Egger test for directional pleiotropy | | |
| --- | --- | --- | --- | --- | --- | --- |
|  | Q | P |  | Intercept | Standard error | P |
| Cigarettes per day | 60.41 | 0.032 |  | -0.004 | 0.388 | 0.384 |
| Lifetime smoking index | 134.4 | 0.032 |  | -0.013 | 1.421 | 0.194 |
| Smoking initiation | 172.4 | 0.476 |  | -0.007 | 0.748 | 0.177 |
| Drinks per week | 80.4 | 0.286 |  | 0.007 | 0.748 | 0.108 |

Supplementary Table 4. MR-PRESSO results

| Exposure | MR-PRESSO | | | | | |
| --- | --- | --- | --- | --- | --- | --- |
|  | OR | 95%CI | P | RSSobs | P value of global test | |
| Cigarettes per day | 1.24 | 0.79-1.93 | 0.36 | 63.44 | 0.04 |  |
| Lifetime smoking index | 1.74 | 0.89-3.43 | 0.11 | 138.13 | 0.03 |  |
| Smoking initiation | 1.55 | 1.04-2.33 | 0.03 | 175.03 | 0.47 |  |
| Drink per week | 0.46 | 0.26-0.81 | 0.009 | 82.89 | 0.29 |  |


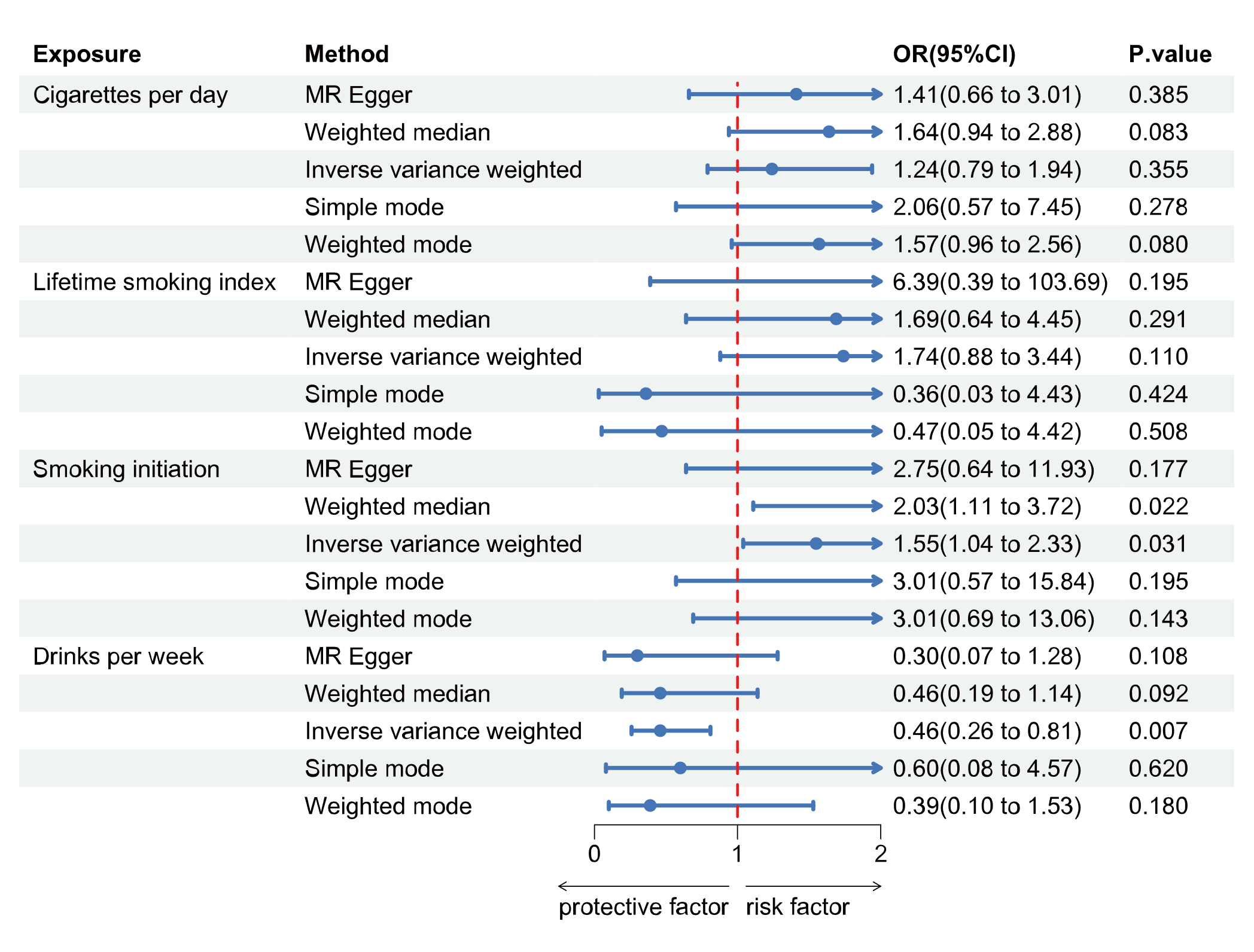


Supplementary Figure 1: Robust methods estimate for the association between smoking, alcohol and renal cell cancer
